# Does the addition of whole-body MRI to routine imaging influence real-world treatment decisions in metastatic breast cancer?

**DOI:** 10.1186/s40644-022-00464-4

**Published:** 2022-06-07

**Authors:** Basrull N. Bhaludin, Nina Tunariu, Nishanthi Senthivel, Amna Babiker, Neil D. Soneji, Nabil Hujairi, Bhupinder Sharma, Sophie E. McGrath, Alicia F. Okines, Alistair E. Ring, Christina Messiou, Kate Downey, Dow-Mu Koh

**Affiliations:** 1 Department of Radiology, The Royal Marsden Hospital, 203 Fulham Rd, London, SW3 6JJ England, UK; 2 Department of Radiology, The Royal Marsden Hospital, Downs Rd, Sutton, SM2 5PT England, UK; 3 Institute of Cancer Research, London, England, UK; 4 Department of Medicine - Breast Unit, The Royal Marsden Hospital, Downs Rd, Sutton, SM2 5PT England, UK; 5 Department of Radiology and Nuclear Medicine, Imperial College Healthcare NHS Trust, Fulham Palace Rd, London, W6 8RF England, UK; 6 Department of Nuclear Medicine, The Royal Marsden Hospital, 203 Fulham Rd, London, SW3 6JJ England, UK; 7 Department of Radiology and Nuclear Medicine, The Royal Marsden Hospital, 203 Fulham Rd, London, SW3 6JJ England, UK; 8 Department of Medicine - Breast Unit, The Royal Marsden Hospital, 203 Fulham Rd, London, SW3 6JJ England, UK

**Keywords:** Whole-body magnetic resonance imaging, Diffusion-weighted imaging, Metastatic breast cancer, Response assessment, Cancer treatment

## Abstract

**Background:**

The assessment of metastatic breast cancer (MBC) can be limited with routine imaging such as computed tomography (CT) especially in bone-only or bone-predominant disease. This analysis investigates the effects of the use of WBMRI in addition to the use of routine CT, bone scintigraphy (BS) and fluorine-18-fluorodeoxyglucose positron emission tomography with computed tomography (FDG-PET/CT) on influencing systemic anti-cancer treatment (SACT) decisions in patients with known MBC.

**Methods:**

MBC patients undergoing SACT who had WBMRI undertaken within 8 weeks of either a routine CT, BS or FDG-PET/CT were reviewed retrospectively. The clinical indications for undertaking the WBMRI examinations were recorded. Data on the extent and distribution of the disease were collected and discordance/concordance of disease status across the imaging modalities were compared. SACT decisions at each time point were also evaluated.

**Results:**

There were 105 MBC patients with 148 WBMRI studies paired with CT, BS or FDG-PET/CT. 50 pairs (33.8%) showed differences in the extent of disease, with 44 pairs due to additional sites (AS) reported on WBMRI alone.

81 patients (Group 1) had one WBMRI paired with routine imaging due to a variety of indications, with clinical symptoms (such as bone pain) being the most common (24.7%).

24 patients (Group 2) had more than one WBMRI study paired with routine imaging comprising 67 pairs. 13/67 pairs (19.4%) showed discordance in assessments. 10/13 pairs had progressive disease (PD) reported on WBMRI alone.

SACT change due to AS reported on WBMRI alone occurred in 21/23 pairs (91.3%) in Group 1. SACT change due to PD reported on WBMRI alone in Group 2 occurred in 6/14 pairs (42.9%). SACT change due to AS/PD in both groups occurred in 11/102 pairs (10.8%) with known invasive ductal carcinoma (IDC) and 13/28 pairs (46.4%) with invasive lobular carcinoma (ILC).

**Conclusions:**

The use of WBMRI in MBC led to earlier recognition of PD and SACT change compared with the other imaging modalities. A higher proportion of discordant response assessments and SACT changes were observed in ILC compared with IDC in our patient group, although larger-scale studies are required to investigate this further.

## Background

Metastatic breast cancer (MBC) is routinely assessed with Computed Tomography (CT) of the thorax, abdomen and pelvis and is the recommended modality of choice for breast cancer staging by the Royal College of Radiologists (RCR) in the UK [1]. Other national and international guidelines such as in the European School for Medical Oncology (ESMO) and National Comprehensive Cancer Network (NCCN) also recommended CT to stage patients with early breast cancer at high risk of having MBC [2, 3]. It is a quick and easily accessible examination and standardised response assessments can be undertaken using Response Evaluation Criteria in Solid Tumours (RECIST) measurements [4], allowing patients to enter clinical trials for novel anti-cancer agents.

Bones are the most common site of metastases in MBC, with about 70% of patients who die from breast cancer having evidence of bone metastases [5]. Bone metastases are predominantly lytic at initial presentation but can also be lytic and sclerotic in combination [6]. The development of sclerotic bone lesions during treatment in the absence of any features of disease progression is recognised as a sign of treatment response by the MD Anderson Cancer Centre criteria [7] but is a common pitfall and can often be mistaken for progressive disease. It is commonly difficult to distinguish between progressive disease or treatment response in patients with bone-only disease as there can be little or no change in the size of the sclerotic bone changes. Furthermore, bone metastases are also considered non-measurable lesions by RECIST 1.1 unless there is a measurable extra-osseous soft tissue component [4], frequently excluding such patients from clinical trials. Due to these reasons, the use of CT for response assessment in MBC particularly in bone-only or bone-predominant disease is very limited.

Bone scintigraphy (BS) using technetium labelled phosphate analogues such as technetium-99 m disodium oxidronate or methylene diphosphonate remains widely used, particularly in the initial staging of MBC. The use of BS for response assessment in bone metastases is limited due to the ‘flare’ phenomenon. A ‘flare’ may be precipitated by treatment response and can often be misinterpreted as disease progression [8]. It is described as an increase in radionucleotide uptake secondary to new bone formation associated with healing metastases rather than progressive disease and is seen typically between 2 and 12 weeks following initiation of treatment however can be seen up to 6 months. The RCR has stated in their guidelines that BS ‘does not perform well in follow-up and for monitoring treatment response’ [1].

Fluorine 18 fluorodeoxyglucose (FDG) positron emission tomography with computed tomography (FDG-PET/CT) is another functional imaging modality that is used to detect the changes in metabolic activity related to the uptake of glucose. It has a higher sensitivity and specificity than BS in the detection of bone metastases in MBC [9–11] and can also show alterations in metabolic activity before any appreciable change in the size of the metastatic lesions is identified [12]. Guidelines from the ﻿European School of Oncology Metastatic Breast Cancer Task Force have stated that the use of FDG-PET/CT in routine disease reassessment of MBC is not recommended due to the lack of sufficient data demonstrating its cost-effectiveness compared to CT and BS [13]. The use of FDG-PET/CT is also limited in invasive lobular carcinoma (ILC), as FDG uptake in bone metastases due to ILC histology disease can be lower (and also ‘false-negative’) compared with invasive ductal carcinoma (IDC) [14, 15].

There is an increasing interest in the use of whole-body magnetic resonance imaging (WBMRI) in the assessment of metastatic bone disease in oncology, particularly in myeloma and metastatic prostate cancer. WBMRI does not involve ionising radiation or intravenous contrast, and this imaging modality enables the differentiation between benign and malignant lesions by combining morphological assessments with measurements of water diffusivity and cellular density [16]. WBMRI is currently accepted as the gold standard in the assessment of myeloma in the UK [17]. Response assessment criteria have also been published in both myeloma and metastatic prostate cancer such as the Myeloma Response Assessment and Diagnosis System (MY-RADS) and Metastasis Reporting and Data System for Prostate Cancer (MET-RADS-P) to allow standardisation in the interpretation and reporting of WBMRI studies [18, 19]. Although there is no similar tool that has been developed for MBC, the RCR guidelines have recommended the use of WBMRI for the evaluation of equivocal findings on other imaging modalities [1].

At our institution, patients with bone-only or bone-predominant MBC are routinely restaged with CT, but since the introduction of WBMRI in 2012, there has been an increased utilisation of WBMRI as an additional tool alongside CT, BS or FDG-PET/CT in MBC assessment. WBMRI is used in a number of scenarios, for example, in cases where the initial imaging shows stable disease but there is clinical suspicion of disease progression, if tumour markers are uninformative or in cases of difficult liver imaging such as pseudocirrhosis.

Previous studies have examined the impact of WBMRI in addition to CT, FDG-PET/CT and BS in influencing systemic treatment decisions [20–22]. For example, a retrospective study of 210 patients with MBC who had their disease evaluated with both CT and WBMRI reported that WBMRI scans detected disease progression in an additional 18.9% of scans [20]. A subsequent prospective study of 45 MBC patients assessing WBMRI compared to bone scintigraphy and CT scans as response evaluation in patients with bone metastases reported that CT and bone scintigraphy each detected approximately half of progressions detected by WBMRI [22]. The aim of this retrospective analysis is to evaluate the effects of the use of WBMRI in addition to the use of routine CT, BS and FDG-PET/CT on influencing systemic anti-cancer treatment decisions in patients with known MBC using real-world data from our own database and to assess how our practice compares with previous published studies.

## Methods

Approval to undertake this analysis as a service evaluation was granted by the Committee for Clinical Research at the Royal Marsden Hospital (reference: SE1070). All patients with known MBC who had undergone WBMRI between 1st May 2012 and 31 January 2021 were screened from our database. All patients with known MBC who had undergone WBMRI and either CT, BS or FDG-PET/CT within 8 weeks of the WBMRI as part of their routine response assessment to systemic anti-cancer therapy (SACT) were eligible for the study. Patients who underwent WBMRI examinations for baseline disease staging for newly diagnosed breast cancer were excluded.

Data were collected from imaging reports of the original WBMRI, CT, FDG-PET/CT or BS from our electronic medical records in order to capture the original decision-making process. The clinical indication of the WBMRI written on the request form was also captured to identify the reason for adding WBMRI to CT, FDG-PET/CT or BS.

The WBMRI examinations had been reported by consultant radiologists with subspecialty interest in oncological imaging, with one to nine years’ experience of reporting WBMRI at the time of producing the reports. Reported sites of disease were documented: bone, visceral/soft tissue (e.g. liver, lungs, nodes, peritoneum) or both. Data were obtained to determine whether there was a difference in the disease extent across the different modalities. If a site of metastatic disease was reported in only one modality, this was regarded as an additional site (AS). For the pairs where a follow-up WBMRI was undertaken subsequently with another CT, FDG-PET/CT or BS, these pairs were considered as part of response assessment. The reports were reviewed to determine whether there was progressive disease (PD), defined as any increase in the extent of disease compared to the baseline examinations. A comparison was made with the paired imaging modality to establish whether there was discordance in the response assessments. If there was such discordance, the AS of disease reported by the modality with PD were documented.

Patient demographics including the histological subtype, tumour grade and receptors were recorded. Oestrogen receptors (ER) and progesterone receptors (PgR) were scored using Allred score, with a score of 3–8/8 considered positive. Human epidermal growth factor receptor 2 (HER2) positive was defined as a 3 + score on immunohistochemistry (IHC), or an IHC 2 + score which was also positive on in situ hybridisation, as per the American Society of Clinical Oncology (ASCO) guidelines [23]. In all cases where AS of disease or discordance in assessments were identified, data were also collected to evaluate whether systemic treatment or other patient management decisions had been changed by the clinical team. This data was documented by the oncology team who were aware of the presence of the additional sites on the WBMRI but the overall disease status written on the WBMRI report were not made available on the data collection spreadsheet.

All WBMRI examinations were performed on a 1.5 Tesla MAGNETOM Aera (Siemens Healthcare, Erlangen, Germany) scanner at the Royal Marsden Hospital. The MRI parameters are summarised in Table 1. The following sequences were used for whole body imaging (from the skull vertex to the mid-thighs): axial whole-body ADC maps and b50, b600 and b900 DWI with three-dimensional coronal maximum intensity projection (MIP) reconstruction of b900 values, axial whole-body T1-weighted Dixon sequences (in- and out-of-phase, water-only and fat-only sequences and, derived relative water- and fat-fraction maps), axial whole-body T2-weighted sequences. The following sequences were used for whole spine imaging: sagittal whole-spine T1-weighed and sagittal whole-spine T2-weighted sequences. CT was undertaken of the chest, abdomen and pelvis with intravenous contrast according to standard local departmental protocol either within the same institution or at the patients’ local hospital. Bone scintigraphy was performed on gamma cameras where patients were imaged 3 hours after injection with 600 Megabecquerel of technetium-99m disodium oxidronate or methylene diphosphonate. FDG-PET/CT was performed after 60 minutes of uptake period post injection with a dose calculated according to body weight as per the European Association of Nuclear Medicine (EANM) guidelines [24]. CT component of FDG-PET/CT was performed as low dose unenhanced study for attenuation correction purposes.

**Table 1:** Parameters for WBMRI

MRI Parameter	Spine imaging		Skull vertex to mid-thighs imaging		
	**T1-weighted**	**T2-weighted**	**T2-** **weighted**	**Gradient-Echo Dixon**	**Diffusion-weighted imaging**
**Imaging sequence**	TSE	TSE	HASTE	3D gradient echo (FLASH)	EPI
**Orientation**	Sagittal	Sagittal	Axial	Axial	Axial
**Number of stations**	2	2	5–6	5–6	5–6
**Slice thickness (mm)**	4	4	5	5	5
**Slices per station**	15–17	15–17	40	40	40
**Slice gap**	10%	10%	0	0	0
**Field of view**	400–440	400–440	380–430	430	430
**Matrix**	384–448	448–512	256–320	160–256	128–134
**Resolution**	1–1.1	1	0.8–1.6	0.8–2.7	1.6–3.4
**Parallel imaging**	2 x (24–38)	2x (30–38)	2x (24–42)	3 × 32 or 2 × 24	2 × 32
**TE (ms)**	11	83–97	82–89	2.38/4.76	64–69
**TR (ms)**	643–683	3110–3790	1000–1400	6.82–7.63	6150–11,500
**Flip angle (degrees)**	150	150	170	3, or 14–20	
**b values (s/mm**^**2**^**)**	-	-	-	-	50, 60, 900
**Fat suppression**	None	None	None	Dixon	STIR
**Breathing instructions**	Free breathing	Free breathing	Breath hold	Breath hold	Free breathing
**Acquisition time per station (min:s)**	0:59 -1:36	1:08–1:22	0:42–0:56	0:16–0:19	3:35—6:25

*EPI* Echo-planar imaging, *FLASH* Fast low angle shot, *HASTE* Half-fourier acquisition single-shot turbo spin echo, *STIR*  Short tau inversion recovery, *TE* Echo time, *TR* Repetition time, *TSE* Turbo spin echo

## Results

One hundred forty-one patients had at least one WBMRI within the specified period. 36 patients were excluded who did not meet the eligibility criteria resulting in a total number of 105 eligible patients. The median age was 58-years-old (range 30 – 89). The tumour characteristics based on the most recently available pathology of the breast cancer patients are summarised in Table 2. A total of 148 WBMRI examinations were paired with another imaging modality: 92 (62.2%) were paired with CT only, 17 (11.5%) were paired with FDG-PET/CT only, 4 (2.7%) were paired with BS only and 35 (23.6%) were paired with both CT and BS. 121 (81.8%) pairs were done within 4 weeks apart, and 27 (18.2%) pairs had the examinations performed between 4 to 8 weeks apart.

**Table 2:** Breast cancer characteristics in our population

Characteristic	N	%
**Histology**		
IDC	77	73.3
ILC	15	14.3
Mixed ductal and lobular	9	8.6
Other	1	1.0
Unknown	3	2.8
**Grade**		
1	3	2.9
2	59	56.2
3	37	35.2
Unknown	6	5.7
**ER**		
Positive	96	91.4
Negative	9	8.6
Unknown	0	0
**PgR**		
Positive	72	68.5
Negative	24	22.9
Unknown	9	8.6
**HER2**		
Positive	13	12.4
Negative	90	85.7
Unknown	2	1.9

The patients were divided into two groups. Group 1 consisted of 81 out of 105 patients (77.1%) who had serial CT or FDG-PET/CT for assessment of known MBC and the addition of WBMRI was used for a number of indications, summarised in Table 3. The most common reasons include clinical symptoms such as bone pain and limb weakness (24.7%), indeterminate findings on other imaging modalities (19.8%) and a rise in tumour markers (9.9%). As there were no previous WBMRI examinations in this group, comparison of response assessments across the different modalities cannot be concluded accurately so a comparison of the disease extent only was made. 20 out of 81 patients in this group had known bone-only disease on CT. 7 (8.6%) of these were deemed difficult to assess with CT which prompted a WBMRI request in order to establish the extent of bone-only disease or to determine disease activity. Group 2 comprised the remaining 24 (22.9%) out of the 105 patients who had at least 2 pairs of examinations undertaken as part of response assessment for MBC, with a total of 67 pairs available for evaluation.

**Table 3:** Indications for WBMRI introduced during the course of MBC re-assessment which were previously assessed with CT or FDG-PET/CT

Indications for WBMRI		N (%)
Clinical symptoms (e.g. bone pain, limb weakness)		20 (24.7)
Indeterminate findings on other imaging modalities		16 (19.8)
Rise in tumour markers		8 (9.9)
Bone-only disease which was difficult to assess with CT		7 (8.6)
Pseudocirrhotic liver		4 (4.9)
Deranged blood results (e.g. anaemia, pancytopaenia, hypercalcaemia)		4 (4.9)
Recommended by radiologist or at the multidisciplinary team meeting		3 (3.7)
Other (e.g. CT/FDG-PET/CT declined by patients due to radiation concerns, clinical trial entry requirements)		7 (8.6)
Combination of indications: Rise in CA 15–3 tumour marker plus:	clinical symptoms	8 (9.9)
	indeterminate findings on other imaging modalities	1 (1.2)
	deranged blood results	2 (2.4)
	recommended by radiologist	1 (1.2)

### Extent of disease

The extent of disease was examined in both groups combined, with a total of 148 pairs. 50 pairs (33.8%) showed differences in the extent of disease between WBMRI and CT, FDG-PET/CT or BS (Tables 4 and 5). There were 32 out of 81 pairs showing differences in disease extent in Group 1, and 18 out of 67 pairs with differences in disease extent in Group 2. Pooling the data from both groups together, 44 (88%) were due to AS reported on WBMRI only compared to the other modalities. 39 of these were AS reported on WBMRI only compared to CT only or CT and BS; 5 were AS reported on WBMRI only compared to FDG-PET/CT – the distribution of the AS on WBMRI compared to the other imaging modalities are detailed in Table 5. The commonest AS only detected with WBMRI was in the viscera/soft tissues, particularly in the liver in 17 (34%), closely followed by the bones in 16 (32%). Out of the 15 patients with ILC, 12 had AS only on WBMRI – 5 had AS in the bones, 4 in the liver, 1 in the peritoneum and 2 in the nodes. No AS in the brain were detected.

**Table 4:** Distribution of AS on all modalities

AS	WBMRI		CT	FDG-PET/CT	BS	WBMRI & CT	WBMRI & FDG-PET/CT	WBMRI & BS
Bone only	16		0	0	1	1	0	1
Bone and viscera/soft tissues	4		0	0	N/A	0	0	N/A
Viscera/soft tissues only	24	Liver 17	Lymph nodes 1	Lungs 1	N/A	0	Retroperitoneal 1	N/A
		Lymph nodes 3						
		Peritoneal 2						
		Liver and peritoneal 2

**Table 5:** Distribution of AS on WBMRI compared with other imaging modalities

AS on WBMRI	Compared to FDG-PET/CT	Compared to CT or CT & BS
Bone only	3	13
Liver only	1	16
Bone and liver	0	4
Peritoneal/retroperitoneal	0	3
Liver and peritoneal	0	2
Nodes	1	1

### Response assessment

**Fig. 1:**
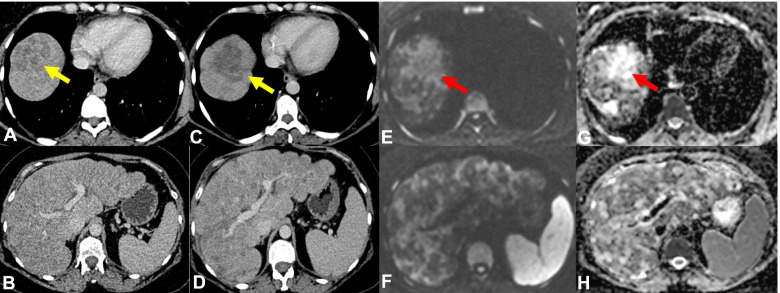
WBMRI better characterising diffuse liver disease. 43-year-old female with patient with ER- PR- positive HER-2 negative metastatic breast cancer to the nodes, lung and liver was being monitored with CT during chemotherapy treatment. Follow up CT following 3 cycles demonstrates enlarging and more confluent liver lesion at the liver dome (**C** – yellow arrow) compared to prior CT (**A**—yellow arrow) and a greater number of lesions elsewhere within the liver suggesting worsening disease (**D**) when compared to prior CT (**B**) however tumour markers were improving on treatment. The increasingly irregular liver contour suggests pseudocirrhosis (**D**). WBMRI was performed to further evaluate the CT findings. The high b value (b900) DWI images (**E**) and corresponding ADC map (**F**) show diffuse disease with some lesions demonstrating high ADC values representing low cellularity/treated disease and others demonstrating low ADC values representing high cellularity/active disease in keeping with a mixture of treated and active disease, indicating a favourable response to treatment in some areas

A comparison of response assessment between WBMRI and either CT, FDG-PET/CT or BS were undertaken for Group 2. Out of the 67 pairs in this group, stable disease (SD) was reported in 47 pairs, partial response (PR) in 6 pairs, and PD in 18 pairs, with discordance in assessments in 13 pairs (19.4%). Out of the 13 pairs with discordance, 10 (76.9%) had PD reported on WBMRI only compared to other modalities and 3 (23.1%) had PR reported on WBMRI only compared to other modalities. 5 pairs (38.5%) had PD in bone disease reported only on WBMRI. PD in viscera/soft tissues were reported only on WBMRI in two cases (16.7%) (one in the liver and another in the retroperitoneum), and 3 (23.1%) had PD of bone and liver disease reported only on WBMRI. There were 2 cases where the CTs were reported as PD due to an increase in the extent of sclerotic bone changes but WBMRI was reported as PR, and 1 case where the CT was reported as PD due to then increase in size of liver metastases on a background of pseudocirrhosis but WBMRI was reported as PR (Fig. 1). None had PD reported only on BS, FDG-PET/CT or CT alone.

### Change in SACT

**Fig. 2:**
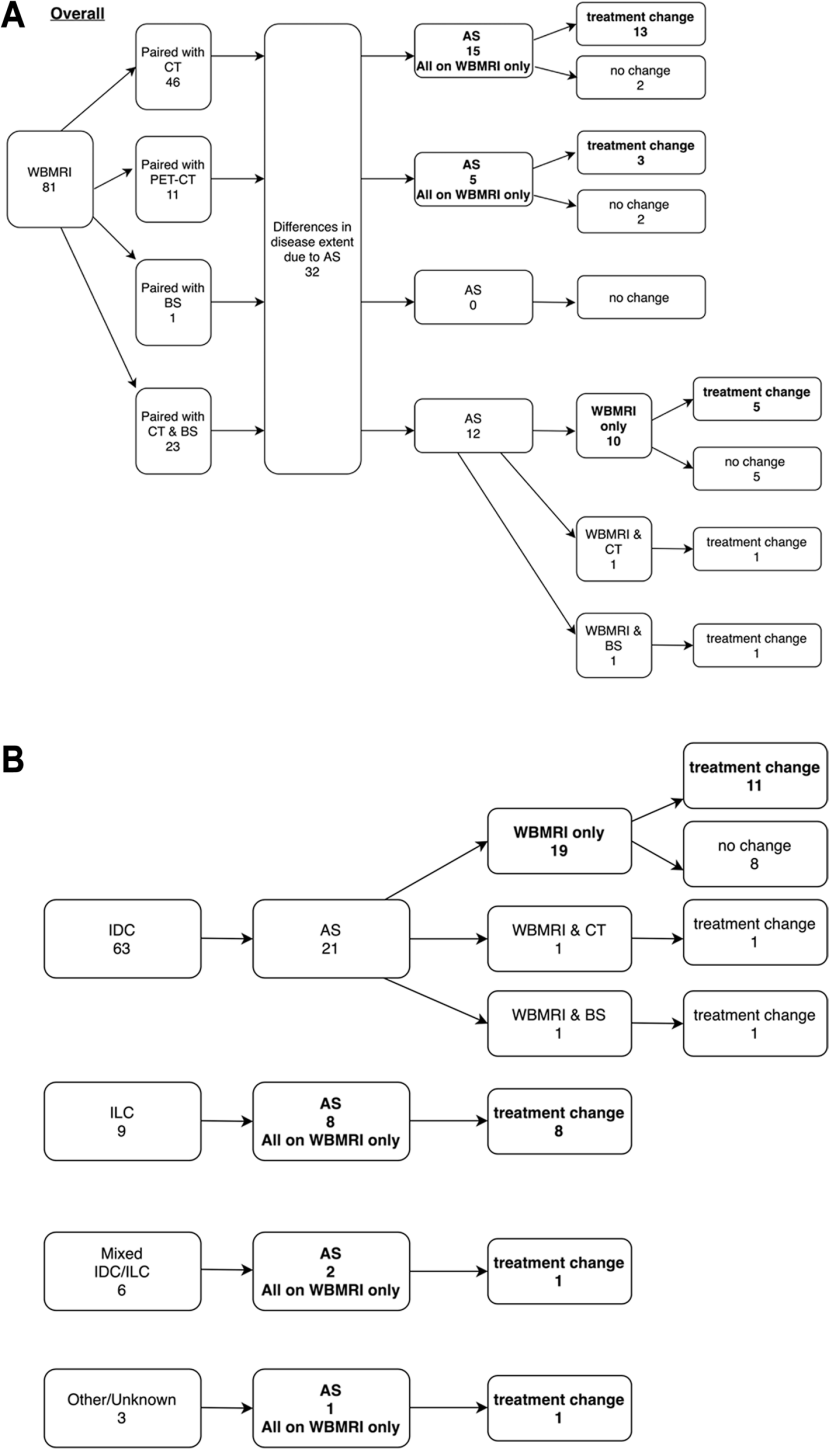
**a** Flowchart showing the different combinations of paired examinations, the distribution of the differences in disease extent and treatment changes in Group 1. **b** Flowchart showing the distribution of additional sites of disease and treatment changes according to histological subtypes in Group 1

A change in SACT occurred in 23 pairs in Group 1 (Fig. 2a and b). This was due to AS reported on WBMRI alone in 21 (91.3%) out of the 23 pairs. When separated into histological subtypes, a change in SACT due to AS reported on WBMRI alone occurred in 11 (17.5%) out of 63 pairs with IDC, 8 (88.9%) out of 9 pairs with ILC, 1 (16.7%) in 6 pairs with mixed IDC and ILC, and 1 (33.3%) in 3 pairs with other/unknown subtypes.

**Fig. 3:**
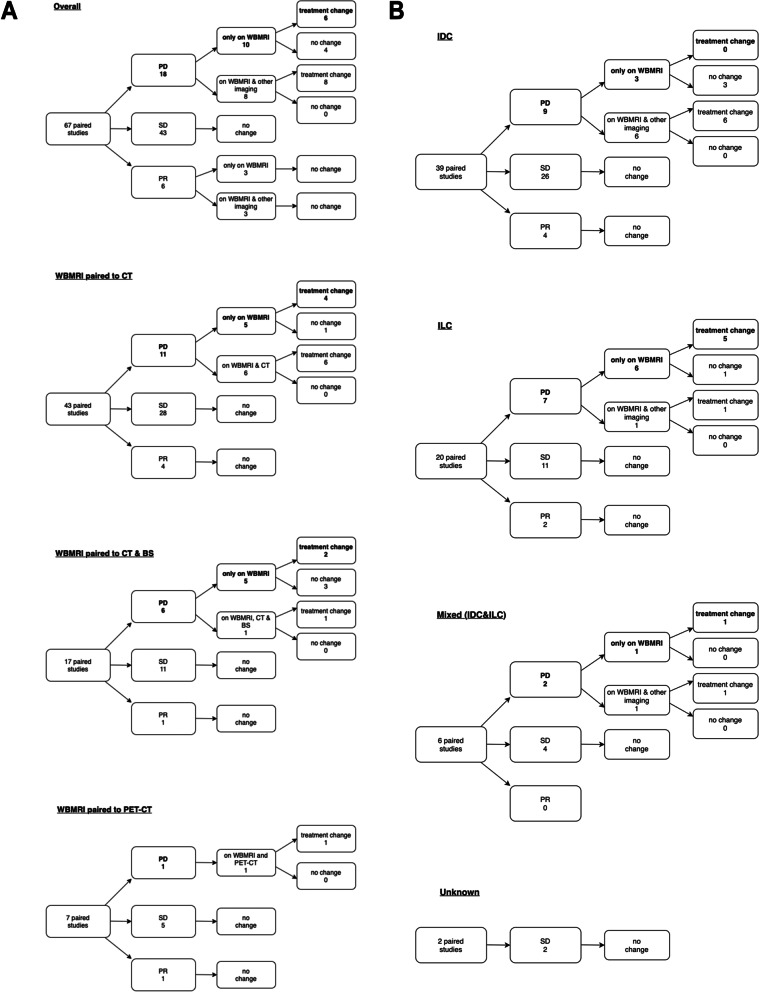
**a** Flowcharts showing the differences in response assessments between the paired imaging modalities and treatment changes in Group 2. **b** Flowcharts showing the differences in response assessments between the paired imaging modalities and treatment changes in Group 2 according to histological subtypes

A change in SACT was made in 14 out of 67 pairs in Group 2 (Fig. 3a and b). The change in SACT was due to PD reported on WBMRI only in 6 (42.9%) out of 14 pairs. When separated into histological subtypes, a change in SACT occurred in patents with ILC due to PD reported only on WBMRI in 5 (35.7%) out of 14 pairs and 1 (7.1%) in mixed IDC and ILC phenotypes. There were no SACT changes due to PD reported on WBMRI in IDC and the unknown phenotypes. All other SACT changes were due to PD reported on both WBMRI and the paired imaging modalities.

**Fig. 4:**
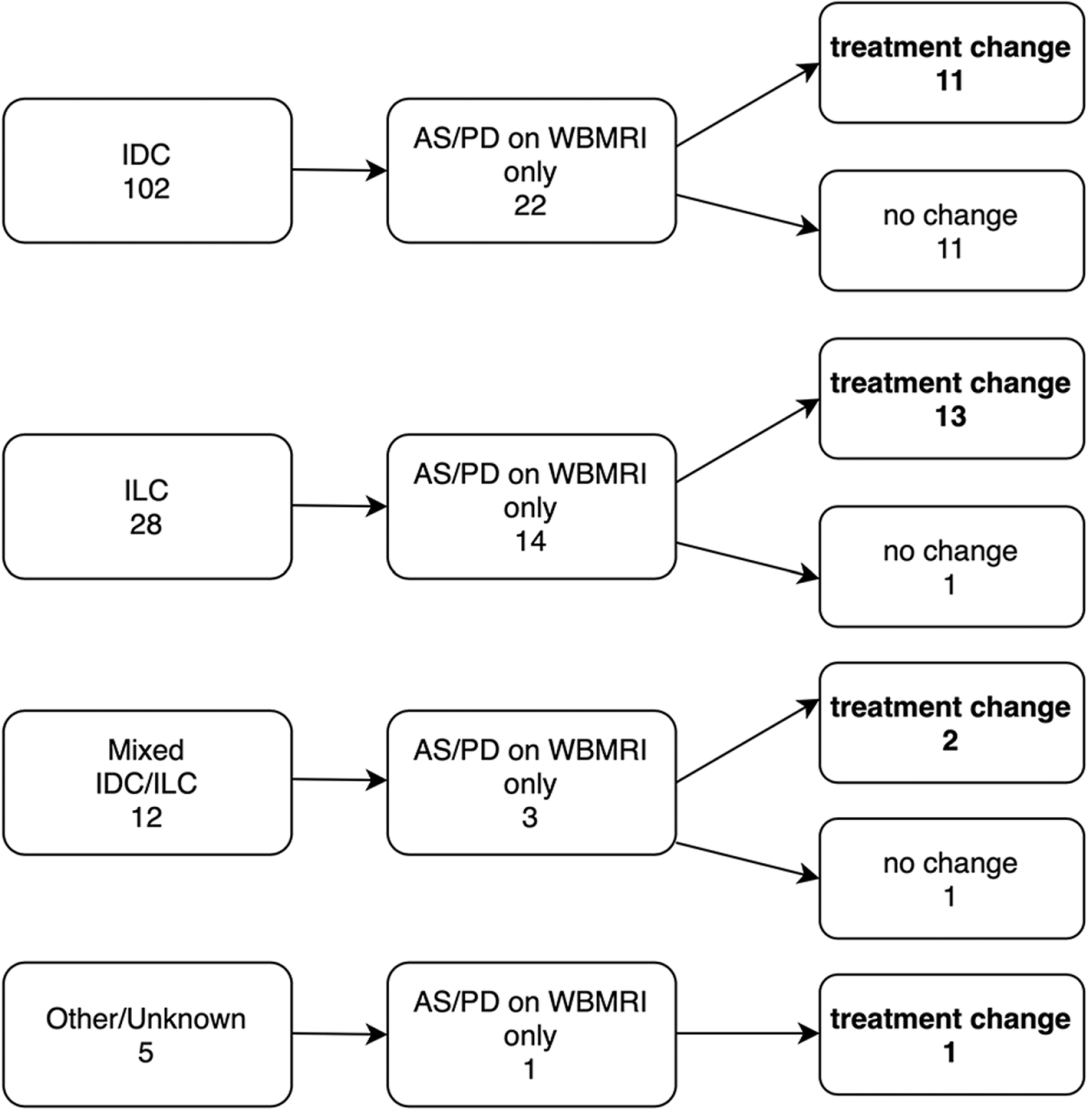
Pooled data on the number of AS/PD reported on WBMRI only and treatment changes according to histological subtypes

Pooling both groups, SACT changes due to AS/PD reported only on WBMRI occurred in 11 (10.8%) out of 102 in IDC pairs, 13 (46.4%) out of 28 in ILC pairs, 2 (16.7%) out of 12 in mixed IDC and ILC pairs, and 1 (20%) out of 5 in the other/unknown subtypes pairs (Fig. 4).

## Discussion

**Fig. 5:**
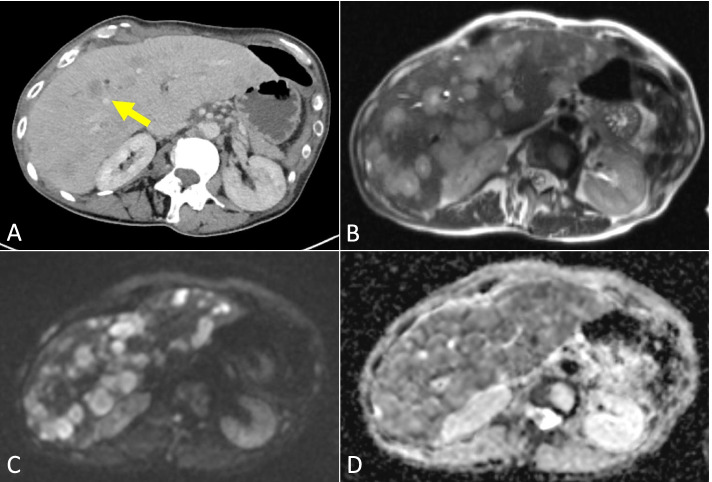
Disease underestimated by CT. 55-year-old female with ER- PR- positive HER-2 negative infiltrating ductal carcinoma with axillary nodal and liver metastases. CT demonstrates bi-lobar liver metastases but significantly underestimates the burden of disease when compared to the WBMRI performed 2 days later. CT (**A**) demonstrates liver metastases measuring up to 18 mm within segment VIII (yellow arrow) however axial T2 (**B**), high b-value (b900) DWI sequences (**C**) and corresponding ADC map (**D**) from the WBMRI study demonstrates many more lesions not appreciated on CT

**Fig. 6:**
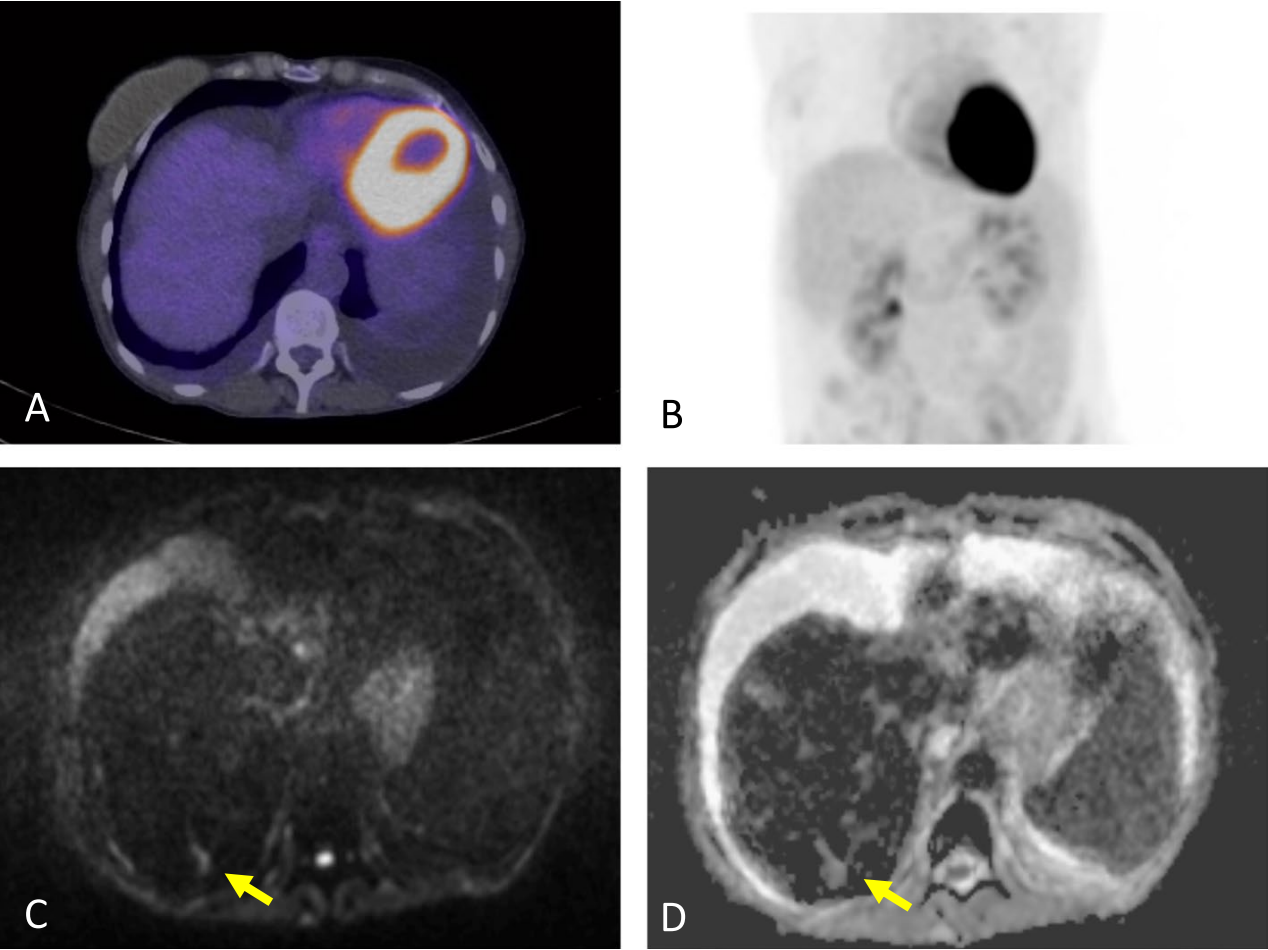
Additional sites of disease on WBMRI compared to FDG/PET-CT. 56-year-old female with ER- PR- positive HER-2 negative infiltrating ductal carcinoma with bone and liver metastases. Worsening liver function tests with decreasing haemoglobin and platelet count were noted whilst on Exemestane. Fused PET-CT images (**A**) and FDG/PET-CT MIP (**B**) showed no significant pathological activity in the liver. There is pseudocirrhosis, ascites and pleural effusions. High b-value (b900) image (**C**) and corresponding ADC map (**D**) of a WBMRI study undertaken four weeks later demonstrated multiple liver metastases measuring up to 20 mm in the posterior right hepatic lobe (yellow arrow) not appreciated on the FDG/PET-CT study

Our results demonstrate that WBMRI identified more sites of disease compared to CT, BS and FDG-PET/CT in approximately one-third of our population. In our study, the most common AS were in the bones and the liver (Figs. 5 and 6), whereas the bone was the most common AS in similar retrospective studies [20, 21]. A prior meta-analysis examining the detection of bone metastases by WBMRI concluded that MRI has a higher pooled sensitivity and specificity compared to CT and BS, and similar to FDG-PET/CT (pooled sensitivity of 89.7%, 72.9%, 90.6% and 86.0% and pooled specificity of 96.8%, 94.8%, 95.4% and 81.4% respectively for FDG-PET/CT, CT, MRI and BS) [25]. Our population had a higher proportion of visceral/soft tissue disease (predominantly of the liver) as an AS detected on WBMRI, likely because our patient population had established MBC and had WBMRI introduced later on in their treatment due to the indications in Table 3 and because of this, it is reasonable to expect that our patients would have more advanced disease compared to the population in the earlier similar studies by Kosmin and Zugni *et. al.* [20, 21].

**Fig. 7:**
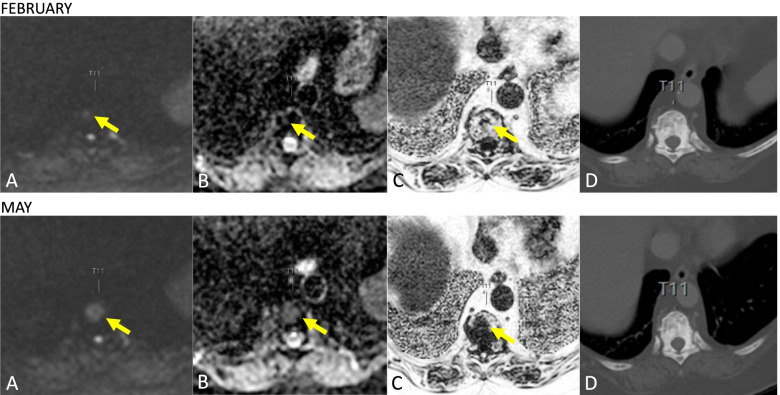
Progressive disease on WBMRI not identified on CT. 80-year-old female with ER- PR- positive HER-2 negative infiltrating ductal carcinoma with bone metastases on first presentation. Tumour marker rise was noted whilst on Letrozole and Denusomab over a three-month period. WBMRI demonstrated clear progression at several sites but no changes were appreciated on CT performed at the same time points. In this figure high b-value (b900) image (**A**), corresponding ADC map (**B**) and derived fat fraction images from the Dixon sequences (**C**) demonstrate an increase in the size of the lesion at T11 with no appreciable change in the mixed lytic and sclerotic disease burden on CT (**D**)

Discordance in the response assessments between WBMRI and CT or FDG-PET/CT were seen in 19.4% of cases with follow-up imaging of both modalities. WBMRI identified PD before CT and/or BS in 76.9% of those cases, and most commonly in the bones (Fig. 7). 14 pairs within the response assessment group (Group 2) resulted in SACT change, of which 10/14 were due to PD only demonstrated on WBMRI. The identification of PD would have been otherwise delayed. Whether or not this had an impact on the patients’ outcome cannot be determined by this retrospective cohort study. Our results are similar to previous studies by Kosmin and Zugni *et. al.*, which reported a change in SACT due to WBMRI-detected PD in 34.7% and 50% of their cases respectively [20, 21]. A more recent prospective study evaluating the assessment of bone-only MBC in 40 patients has also demonstrated that WBMRI enabled earlier identification of PD before CT and BS in 67% of their cases, with an even poorer performance of BS, which detected PD in only 50% of their cases [22]. The data from these studies and our results indicate that the use of WBMRI in bone-only or bone-predominant MBC is valuable in response assessment by identifying PD earlier.

In our patients with known MBC who are already undergoing surveillance with CT or FDG-PET/CT, about one-half of these patients had an WBMRI due to clinical symptoms, indeterminate findings on imaging or rising tumour markers which warranted further investigation. We found that the use of WBMRI for this setting led to a change in systemic therapy in 91.3%. This supports the RCR guidelines in the use of WBMRI for the evaluation of cases where there are equivocal findings on other imaging modalities [1] and our results show that WBMRI has made an important impact in decisions on whether to continue or change systemic therapy.

**Fig. 8:**
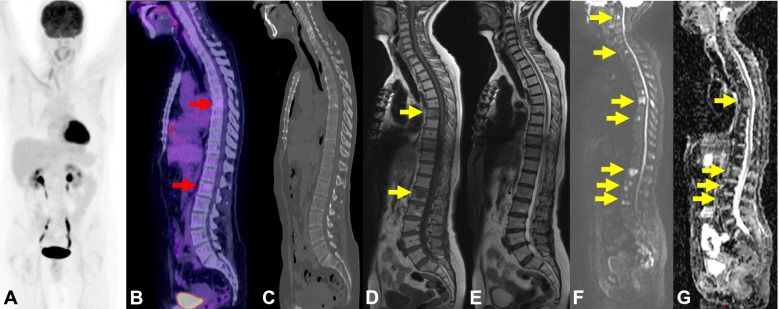
FDG PET-CT and WB MRI in lobular breast cancer. 62-year-old female with a history of treated lobular breast cancer with no history of metastatic disease presented with back pain. FDG PET-CT MIP (**A**) showed no significant or high grade pathological activity. The fused PET-CT images (**B**) showed minimal activity in a few bone lesions (red arrows) although the lesions were barely appreciated on the low-dose CT component (**C**). These were indeterminate but suspicious in this context. WBMRI was performed for further evaluation which showed a greater number of bone lesions (yellow arrows) better seen on Sagittal T1-weighted images (**D**) compared with the Sagittal T2-weighted images (**E**). The bone lesions demonstrate restricted diffusion with high signal on b900 DWI images (**F**) and low signal on ADC map (**G**) in keeping with bone metastases

Our analysis has also shown that a higher proportion of patients with pure ILC had more AS of disease detected on WBMRI compared to other imaging modalities and a higher number of discordant assessments leading to a change of SACT. The pooled data in our results show SACT change in 46.4% in the ILC population compared with 10.8% in the IDC population due to discordant findings between WBMRI and the other imaging modalities. This is lower than reported in the study by Zugni *et. al.,* which showed SACT change in 70% of their patients with ILC due to PD only reported on WBMRI, possibly because they had a larger population of ILC patients (32% compared with 14.3% in our series) [21]. The higher proportion of discordance in assessment of disease extent and response leading to SACT change in ILC is not unexpected and may be due to the infiltrative nature of ILC which tends to form in a single-file (also known as an “Indian-file”) pattern as a result of the pathognomonic loss of the cell adhesion molecule, E-cadherin, compared with IDC [26]. Although the commonest site of metastases for ILC is the bones, with 81% reported on a retrospective review of CT imaging of 57 patients, ILC also tends to spread to unusual sites such as the peritoneum and gastrointestinal tract [27]. The infiltrative nature and unusual pattern of spread of ILC can be difficult to detect on CT compared to IDC. Furthermore, ILC can also show lower FDG uptake on FDG-PET/CT compared to IDC, which reduces the value of FDG-PET/CT in the assessment of ILC [14, 15] (Fig. 8). Given the challenges of imaging metastatic ILC with CT and FDG-PET/CT, WBMRI may be better suited to assess the extent of disease and treatment response.

There are several limitations to this study. The analysis was performed retrospectively which introduces inherent biases. The imaging was not re-reviewed and the data was collected based on the reports to replicate real-world clinical decision making. As a result, some lesions may not have been reported or could have been missed. Furthermore, in routine clinical practice, it is common for radiologists to refer to the other imaging modalities, introducing further biases in reporting and data collection, particularly of the AS (for example, some subtle bone or liver lesions may have not been detected on CT but was reported as a FDG-PET/CT or WBMRI was performed earlier showing more extensive disease). It was also not possible to obtain histopathological proof that all the AS detected on WBMRI represented true metastatic lesions. It is our routine clinical practice to follow-up any suspicious but equivocal lesions to monitor for any interval change, rather than changing treatment on the basis of this, unless clinically indicated.

At our institution, we did not routinely perform both WBMRI and CT/BS/FDG-PET/CT concurrently on all MBC patients. Therefore, we have a smaller number of paired studies compared to the study conducted by Kosmin *et. al.*, even though our patient population is larger [20]. This is because in clinical practice, most of our patients will continue to have response assessments of one modality only (either CT or WBMRI, depending on which provided the most informative data) in order to ensure we are utilising our scanning capacity efficiently, resulting in missing timepoints and fewer paired studies. For this analysis, we chose to include paired modalities up to 8 weeks apart, similar to the study undertaken by Zugni *et. al.*, but this is a longer interval compared to the study undertaken by Kosmin *et. al.* where the interval was only 2 weeks [20, 21]. Due to the longer interval in our study, there could be some degree of progression or treatment response which may have taken place between the different studies, potentially leading to discordant results. However, only 27 out of the 148 pairs (18.2%) had an interval between the WBMRI and another imaging modality of more than 4 weeks apart. Given the retrospective nature of this study, interval of timepoints between the paired studies were not consistent and would not be as tightly controlled within a short interval that could be achieved in a prospective study, but this depicts real-world clinical practice. WBMRI is also a relatively new choice of imaging at our institution, so the adoption of this new modality was variable during the early stages. The variability in imaging approaches for therapy monitoring in MBC may be a reflection of the RCR guidelines which recommends CT in most cases, with utilisation of FDG-PET/CT or MRI for only problem-solving [1]. Furthermore, the ESMO guidelines do not state any specific imaging modalities of response assessment as there is no evidence of overall survival benefit over another [3]. Due to these reasons, there may be some cases where several weeks would have elapsed when a decision is made to proceed with a WBMRI following a multidisciplinary team discussion and by the time the WBMRI examination is scheduled.

It is unclear from the studies conducted so far, including our own, whether the use of WBMRI can lead to better patient outcomes, quality of life and overall survival. Larger scale and randomised studies would be required to examine this. Future work such as the development of a standardised response assessment criteria for MBC would allow patients with bone-only or bone-predominant disease to enter future clinical trials using WBMRI as the imaging modality of choice to provide a more reliable and accurate response assessment of their disease status. Given the higher rate of discordance seen in ILC, a large study examining the utility of WBMRI in the assessment of metastatic ILC is warranted, to determine whether WBMRI would be a better imaging tool in this setting compared to standard CT/BS or other functional imaging modalities such as FDG-PET/CT.

## Conclusions

We have demonstrated that the use of WBMRI in MBC led to earlier recognition of PD and enabled earlier cessation of ineffective SACT in a real-world setting. We have also found a higher proportion of discordant response assessments and SACT changes in ILC compared with IDC although a larger scale study would be required to confirm this observation.

## Data Availability

The datasets used and/or analysed during the current study are available from the corresponding author on reasonable request.

## Abbreviations

AS: Additional site(s)
ASCO: American Society of Clinical Oncology
BS: Bone scintigraphy
CT: Computed tomography
EANM: European Association of Nuclear Medicine
ESMO: European School for Medical Oncology
ER: Oestrogen receptor
FDG: Fluorine 18 fluorodeoxyglucose
HER2: Human epidermal growth factor receptor 2
IDC: Invasive ductal carcinoma
IHC: Immunohistochemistry
ILC: Invasive lobular carcinoma
MBC: Metastatic breast cancer
MET-RADS-P: Metastasis Reporting and Data System for Prostate Cancer
MIP: Maximum intensity projection
MY-RADS: Myeloma Response Assessment and Diagnosis System
NCCN: National Comprehensive Cancer Network
PET: Positron emission tomography
PD: Progressive disease
PgR: Progesterone receptor
PR: Partial response
RCR: Royal College of Radiologists
RECIST: Response Evaluation Criteria in Solid Tumours
SACT: Systemic anti-cancer therapy
SD: Stable disease
WBMRI: Whole-body magnetic resonance imaging
